# Effects of *Lactobacillus acidophilus* KLDS1.0901 on Proliferation and Apoptosis of Colon Cancer Cells

**DOI:** 10.3389/fmicb.2021.788040

**Published:** 2022-02-11

**Authors:** Yingxue Yue, Song Wang, Jialu Shi, Qinggang Xie, Na Li, Jiaqi Guan, Smith Etareri Evivie, Fei Liu, Bailiang Li, Guicheng Huo

**Affiliations:** ^1^Key Laboratory of Dairy Science, Ministry of Education, Northeast Agricultural University, Harbin, China; ^2^Food College, Northeast Agricultural University, Harbin, China; ^3^Heilongjiang Feihe Dairy Co., Ltd., Qiqihaer, China; ^4^Department of Animal Science, Faculty of Agriculture, University of Benin, Benin City, Nigeria; ^5^Department of Food Science and Human Nutrition, Faculty of Agriculture, University of Benin, Benin City, Nigeria

**Keywords:** *Lactobacillus acidophilus*, colon cancer, HT-29 cell, proliferation, apoptosis

## Abstract

Colon cancer is the most common type of malignant tumor. The cytotoxicity effect of lactic acid bacteria may be active by inhibiting cancer cell proliferation, producing anticancer compounds, and inducing apoptosis in cancer cells, but the mechanism is unclear. Our previous study revealed that *Lactobacillus acidophilus* KLDS1.0901 has good probiotic properties. In this study, We screened out the highest inhibition rate of *L. acidophilus* KLDS1.0901 and assessed the effects on the proliferation of HT-29, Caco-2, and IEC-6 cells. Then, the apoptosis mechanism of HT-29 cells was studied when treated with *L. acidophilus* KLDS1.0901. Results showed that *L. acidophilus* KLDS1.0901 inhibited the proliferation of HT-29 and Caco-2 cells in a dose-dependent manner and reached the maximum under the condition of multiplicity of infection (MOI) = 100 (rate of *Lactobacillus* to cells) at 48 h. With the increase in time and MOI, reactive oxygen species in HT-29 cells, the apoptosis rates of HT-29 cells were increased, and the amount of blue fluorescence of the cells was also increased after Hoechst 33258 staining. Furthermore, *L. acidophilus* KLDS1.0901 reduced the mitochondrial membrane potential of HT-29 cells. Notably, 1,133 differentially expressed genes were screened by transcriptomics research, including 531 up-regulated genes and 602 down-regulated genes. These genes were involved in the nuclear factor κB and PI3K-AKT signaling pathways related to the apoptosis of HT-29 cells. These findings suggested that *L. acidophilus* KLDS1.0901 has the potential to be used in the development of a new type of functional foods for adjuvant treatment of colon cancer.

## Introduction

Colon cancer is the fourth leading cause of cancer death globally, with more than one million cases diagnosed each year worldwide, and it is a multifactorial disease involving genetic, environmental, and lifestyle risk factors (Ferlay et al., 2015). The etiology of colon cancer is numerous, including a high-fat diet, obesity, alcohol consumption, chronic inflammation of the gastrointestinal tract, and chronic constipation (Bian et al., 2004). In addition to diet, probiotics are the most common substances used to maintain a healthy microbial community (Balakrishnan and Floch, 2012). The gut microbiota becomes more important in health and disease, and more research has been on regulating the intestinal microbiome and its interaction with the host. 5-Fluorouracil (5-FU) and oxaliplatin are the most effective drugs for the treatment of colon cancer. However, these treatments are expensive and have some side effects. The most common side effects include destroying healthy cells and inducing resistance in cancer cells. In addition, there are side effects such as diarrhea, shrinkage of the intestines, and inability to absorb nutrients effectively (Kakde et al., 2011). Several clinical trials provide strong evidence for probiotics as a supplement treatment strategy. A randomized trial conducted on a total of 398 subjects containing both men and women has shown that there is a lower rate of appearance of moderate and higher atypia graded tumors when taking *Lactobacillus casei*. It suggested the prophylactic implication of probiotics in colorectal cancer (Ishikawa et al., 2005). When subjects took a combination of strain-specific probiotic (microbial cell preparation) and omega-3 fatty acid, the overall quality of life improved, and chemotherapy’s side effects were reduced (Golkhalkhali et al., 2018). Although the results of clinical trials are very encouraging, the number of trials related to this field is still few. A randomized clinical trial with larger sample size, proper randomization process, proper analysis, and verification must be conducted to create more comprehensive and specific evidence to support the theory.

Probiotics are defined as “active microorganisms that are beneficial to the host when they reach a certain number.” The global market share of probiotics could reach US $46.55 billion by 2020 (O’Toole et al., 2017). *Lactobacillus* is a group of food-grade microorganisms generally considered safe in food. *Lactobacillus* is a group of food-grade microorganisms generally considered safe in food. Probiotics have been recommended to treat inflammation and infectious and neoplastic diseases (Dasari et al., 2017). It has been found that probiotics have anticancer effects. Probiotics exert anticancer effects by facilitating anticancer compounds, enhancing the immune system, improving the intestinal barrier, inhibiting cancer cell proliferation, and inducing apoptosis in cancer cells (Heydari et al., 2019).

Meanwhile, the potential mechanisms of probiotics responsible might be altering intestinal microecosystem, lowering intestinal pH, and altering tumor metabolism [e.g., producing short-chain fatty acid (SCFA), conjugated fatty acid] (Chen et al., 2012). Synbiotic combination of *Bifidobacterium lactis* and resistant starch in the rat-azoxymethane model has been proven to protect against the development of clone cancer, which was correlated with increased SCFA production (Le Leu et al., 2010). *Lactobacillus* have various probiotic functions, including cholesterol-lowering activity, antioxidant activity, anticancer activity, immunomodulatory activity, and inhibition of pathogenic bacteria activity. Their probiotic characteristics are attributed to their bile resistance and ability to adhere to the gut microbiota, which allows them to inhibit pathogenic bacteria and make the host healthy (Dixit et al., 2013). Meanwhile, *Lactobacillus* exerts resistance to colon cancer in various forms, such as live and deadly strains, strain components, and metabolites of live strain. The apoptosis of colon cancer cells is due to the activation of cysteine aspartic protease by live lactic acid bacteria (LABs), which inhibits the proliferation of colon cancer cells such as Caco-2 (Thirabunyanon et al., 2009). In a previous study, *Lactobacillus acidophilus* KLDS1.0901 had a strong resistance to acid and bile salt, antimicrobial properties, and high cell adhesion activities (Du et al., 2016). Some experimental studies demonstrated the efficacy of probiotics in cancer prevention and treatment in human and mouse models. Celebioglu et al. (2021) investigate the effects of lime honey on well-known probiotic bacteria *L. acidophilus* LA-5 and *Lactobacillus rhamnosus* GG, and *in vitro* cytotoxic effects of the combination on breast and colon cancer cells MCF-7 and Caco-2. The results showed that adding lime honey to the growth of lactobacilli enhanced cytotoxicity in breast and colon cancer cells. Shida and Nomoto found that *L. casei* Shirota exerts anticancer activity by immunomodulating effects of macrophages, natural killer cells, and T cells on cancer cells (Shida and Nomoto, 2013). Baldwin et al. (2010) found that live *L. acidophilus* and *L. casei* could increase the apoptosis-induction capacity of 5-FU, suggesting that these probiotics may have apoptosis-induction activity in cancer cells.

The imbalance between free radical formation and antioxidants can lead to DNA damage and mutations, increasing cancer incidence. Therefore, antioxidants may play a role in preventing or treating cancer and might induce cell proliferation induction to affect cancer (Elfahri et al., 2016). It has been suggested that probiotics can effectively reduce the carcinogenic chemicals caused by oxidative DNA damage *in vitro* and *in vivo* (Zhang et al., 2013). Experimental studies exhibited that oral administration of specific probiotics and their metabolites reduced the risk of reactive oxygen species (ROS) accumulation and also degraded superoxide anions and hydrogen peroxide (Tsai et al., 2010). Moreover, mitochondrial membrane potential changes in apoptotic cells. Studies demonstrated that apoptosis could be regulated by regulating mitochondrial transmembrane potential (Hofmann et al., 2009).

Therefore, the purpose of this study is to investigate the cytotoxicity effects and its mechanism of *L. acidophilus* KLDS1.0901 in HT-29 cells. *L. acidophilus* KLDS1.0901 has been reported to have probiotic activity (antioxidant properties) and adhesion and to inhibit the proliferation of HT-29 cells. At the same time, the ability of *L. acidophilus* KLDS1.0901 to tolerate gastrointestinal juice can reach more than 80% (Yue et al., 2020). However, there is no report of *L. acidophilus* KLDS1.0901 on its cytotoxicity effects. Notably, we determined the effects of *L. acidophilus* KLDS1.0901 on apoptosis-related signaling pathways in HT-29 cells, which provide a theoretical basis for developing functional *Lactobacillus* with the effect of preventing colon cancer. At the same time, the experiments *in vitro* have laid the foundation for studying the inhibitory effect of *Lactobacillus* on colon cancer *in vivo*.

## Materials and Methods

### Bacterial Strain and Culture

*L. acidophilus* KLDS1.0901, *L. acidophilus* KLDS1.0902, *Lactobacillus plantarum* KLDS1.0317, *L. plantarum* KLDS1.0318, *L. plantarum* KLDS1.03844, *L. plantarum* KLDS1.0386, *L. plantarum* 1-2, *L. rhamnosus* GG, *L. rhamnosus* KLDS1.0205, *L. rhamnosus* 5, *L. plantarum* 1-5, *L. plantarum* 2-2, *L. plantarum* 4-4, *L. plantarum* 4-5, and *L. plantarum* 8-6 were stored in the Key Laboratory of Dairy Science of Northeast Agricultural University. The strains were anaerobically incubated in MRS (De Man, Rogosa, and Sharpe) broth (Hopebio Company, Qingdao, China, HB0384-5) at 37°C for 18 h and subcultured twice before the experiment. Following incubation, the strains were harvested by centrifugation (6,000*g* for 10 min at 4°C) and washed three times with phosphate-buffered saline (PBS) (pH 7.4) buffer. The strains were resuspended to cell culture medium (no penicillin–streptomycin solution).

### Cell Culture

HT-29 cells were purchased from Qingqi Biological Co. Ltd. of Shanghai. Caco-2 cells were obtained from m the American Type Culture Collection (HTB-37, Manassas, VA, United States). IEC-6 cells were purchased from Meisen Cell Technology Co. Ltd. of Zhejiang. High-glucose Dulbecco modified eagle medium (DMEM) cell culture medium supplemented with 10% fetal bovine serum (FBS) and 1% antibiotics (100 U/mL penicillin and 0.1 mg/mL streptomycin) was used to culture three cells. Meanwhile, high-glucose DMEM cell culture medium (no penicillin–streptomycin solution) supplemented with 10% FBS was used to culture cells treated with *L. acidophilus* KLDS1.0901. Cell suspensions were centrifuged at 1,000 revolutions/min (rpm) for 5 min. The cell pellets were resuspended, plated onto 25-cm^2^ culture flask, and grown in a cell culture incubator with 5% CO_2_ at 37°C. The cell culture medium was changed to remove nonadherent cells after 24 h, whereas the media was changed according to the culture conditions. When cells occupied 80% of the culture flask, they were washed with PBS and trypsinized using trypsin–EDTA for expansion and follow-up experiments.

### Cytotoxicity Assay

The inhibition and viability rate were measured by Cell Counting Kit 8 (CCK-8) assay. HT-29 cells were plated in 96-well plates at 1 × 10^4^ cells/well and incubated for 24 h. The HT-29 cells were treated with *L. acidophilus* KLDS1.0901, *L. acidophilus* KLDS1.0902, *L. plantarum* KLDS1.0317, *L. plantarum* KLDS1.0318, *L. plantarum* KLDS1.03844, *L. plantarum* KLDS1.0386, *L. plantarum* 1-2, *L. rhamnosus* GG, *L. rhamnosus* KLDS1.0205, *L. rhamnosus* 5, *L. plantarum* 1-5, *L. plantarum* 2-2, *L. plantarum* 4-4, *L. plantarum* 4-5, and *L. plantarum* 8-6 [multiplicity of infection (MOI) = 10] for 48 h to select the strain of the max inhibition rate. *L. rhamnosus* GG served as the positive control strain, and *L. rhamnosus* 5 as the negative control strain. After the cells culture were completed, the cells were washed three times with PBS of 100 μL, and 110 μL of the mixed CCK-8 solution (100 μL of high-glucose DMEM medium + 10 μL of CCK-8 solution) was added to each well of the plate. The 96-well plates were incubated in an incubator for 1 to 4 h. The absorbance at 450 nm was measured using a microplate reader.


$$\mathrm{Inhibition}\mathrm{rate}\%\frac{\mathrm{Ac}-\mathrm{As}}{\mathrm{Ac}-\mathrm{Ab}}100$$

As is the absorbance of the experimental well (absorbance of cells containing cells, medium, CCK-8, and test compound); Ab is blank hole absorbance (absorbance of wells containing medium and CCK-8); Ac is the control well absorbance (absorbance of wells containing cells, medium, and CCK-8).

HT-29, Caco-2, and IEC-6 cells were plated in 96-well plates at 1 × 10^4^ cells/well and incubated for 24 h. The HT-29, Caco-2, and IEC-6 cells were treated with *L. acidophilus* KLDS1.0901 (MOI = 1, 10, 50, and 100) for 12, 24, and 48 h, respectively. 5-FU (100 μg/mL) served as positive controls. HT-29 cells were plated in 96-well plates at 1 × 10^4^ cells/well and incubated for 24 h. The HT-29 cells were cultured with DMEM cell culture medium of pH 6.8 and pH 7.4 for 48 h, and pH 7.4 as the control group. The detection method was the same as above.

### Morphology Analysis of HT-29 Cell Apoptosis

HT-29 cells were plated in 6-well plates at 2 × 10^5^ cells/well and incubated for 24 h. The cells were treated with *L. acidophilus* KLDS1.0901 (MOI = 1, 10, 50, and 100) for 24 and 48 h, respectively. 5-FU (100 μg/mL) served as positive controls. The culture solution was sucked out, and 0.5-mL fixative was added for 10 min in 6-well plates. The fixative was removed and washed twice with PBS of 1 mL for 3 min each time. Hoechst 33258 staining solution was added to 0.5 mL and stained for 5 min. The staining solution was removed, washed twice with PBS of 1 mL for 3 min each time, and drained the liquid. A drop of antifluorescence quenching liquid was dropped on 6-well plates and observed under a fluorescence microscope.

### HT-29 Cell Apoptosis Analysis

The treatment method in HT-29 cell was the same as the morphology analysis of HT-29 cell apoptosis. The cells were collected in a 15-mL centrifuge tube and washed from 6-well plates once with PBS of 1 mL. The cells were digested with the appropriate amount of trypsin cell digesting solution for 2 min; the appropriate amount of serum-containing high-glucose DMEM cell culture medium was added to terminate the digestion, pipetted the cells gently, and transferred to a 15-mL centrifuge tube. The cells were centrifuged at 1,000 rpm for 5 min, the supernatant was discarded, the cells were resuspended gently by adding 195 μL of annexin V–fluorescein isothiocyanate (FITC) binding solution, and 5 μL of annexin V–FITC mix was gently added. Then, 10 μL of propidium iodide stain was added and mixed gently. The cells were incubated at room temperature (20°C–25°C) for 10–20 min in the dark, then put in centrifuge tubes in ice, and detected by flow cytometry immediately.

### Measurement of Reactive Oxygen Species and Mitochondrial Membrane Potential in HT-29 Cells

HT-29 cells were plated in 6-well plates at 2 × 10^5^ cells/well and incubated for 24 h. The cells were treated with *L. acidophilus* KLDS1.0901 (MOI = 1, 10, 50, and 100) for 24 and 48 h, respectively. The cells were collected in a 15-mL centrifuge tube, centrifuged at 1,000 rpm for 5 min, washed twice with PBS of 1 mL, and washed once with a serum-free cell culture medium of 1 mL. The cells were suspended in an appropriate volume of diluted DCFH-DA and incubated in a cell incubator of 37°C for 20 min. They were then washed three times with a serum-free cell culture medium to remove DCFH-DA that did not wholly enter the cells. The cells were suspended in PBS of 1 mL, and 100 μL was placed in a 96-well plate. The experiment was performed in parallel three times. An excitation wavelength of 488 nm and an emission wavelength of 525 nm was used and detected by a fluorescence spectrophotometer.

All cells were washed once with PBS of 1 mL, then 1 mL high-glucose DMEM cell culture medium and 1 mL JC-1 dyeing working solution were mixed thoroughly and incubated for 20 min at 37°C in a cell culture incubator. The supernatant was aspirated after being incubated at 37°C; the supernatant was aspirated, then washed twice with JC-1 staining buffer (1×), and added with 2 mL of high-glucose DMEM cell culture medium observed under a fluorescent microscope.

For detection with a fluorescence spectrophotometer, the cells were collected and resuspended in a medium of 0.5 mL of high-glucose DMEM cell culture medium, then 0.5 mL of JC-1 staining solution was added and mixed several times and incubated in a cell incubator at 37°C for 20 min. The cells were centrifuged (600*g* at 4°C) for 3 to 4 min and then washed twice with JC-1 staining buffer (1×). Finally, the cells were resuspended in an appropriate amount of JC-1 staining buffer (1×) and detected by a fluorescence spectrophotometer.

### RNA Extraction and Library Preparation for RNA-Seq

HT-29 cells were grown in a plate of Φ6 at 5 × 10^5^ cells/plate and incubated for 24 h. The cells were treated with *L. acidophilus* KLDS1.0901 (MOI = 100) for 48 h. According to the manufacturer’s instructions, total RNA was isolated from HT-29 cells using the TRNzol reagent (Invitrogen, United States). Three micrograms of RNA per sample was used as input material for the RNA sample preparations. Sequencing libraries were generated using NEBNext^®^ Ultra™ RNA Library Prep Kit for Illumina^®^ (#E7530L, NEB, United States) following the manufacturer’s recommendations, and index codes were added to attribute sequences to each sample. Briefly, mRNA was purified from total RNA using poly-T oligo-attached magnetic beads for eukaryote. (For prokaryote, rRNA was removed from total RNA using Ribo-Zero rRNA Removal Kit to purify mRNA.) The mRNA is prepared as short fragments and mixed with the fragmentation buffer. First-strand cDNA was synthesized using random hexamer primer and RNaseH. Second-strand cDNA synthesis was performed using a buffer, dNTPs, DNA polymerase I, and RNase H. The library fragments were purified and resolved with EB buffer, and then a terminal repair, A-tailing, and adapter added were implemented. After size selecting and retrieving by AMPure XP beads, the products were used as the index polymerase chain reaction (PCR) templates.

RNA library concentration was measured using Qubit^®^ RNA Assay Kit in Qubit^®^ 3.0 to preliminarily quantify and then dilute to 1 ng/μL. Insert size was assessed using the Agilent Bioanalyzer 2100 system (Agilent Technologies, CA, United States), and suitable insert size was accurate quantification using StepOnePlus™ Real-Time PCR System (Thermo Fisher Scientific, Waltham, MA, United States) (Library valid concentration >10 nM). The library could be sequenced using Illumina HiSeqTM X TEN.

### Gene Ontology and Kyoto Encyclopedia of Genes and Genomes Enrichment Analyses for Differentially Expressed Genes

The Gene Ontology (GO) enrichment analysis of differentially expressed genes (DEGs) was implemented using the GO seq R package, which was also used to correct gene length bias. DEGs were considered to be significantly enriched in GO categories that had a corrected *p* < 0.05. We used KOBAS software to test the enrichment of DEGs in the Kyoto Encyclopedia of Genes and Genomes (KEGG) pathway statistically.

### Protein–Protein Interaction Network Construction

The protein–protein interaction (PPI) network, which was experimentally validated, was constructed based on the overlapping genes between DMGs and DEGs. The STRING database^1^ was used to search for predicting PPIs. DEGs were mapped into PPIs in the present study, and a combined score > 0.9 was used as the cutoff value. The closest 150 edges were constructed using Cytoscape 3.0.0.

### Real-Time Quantitative Polymerase Chain Reaction

The relative expressions of 10 genes were determined by quantitative PCR (qPCR), and β-actin was selected as a housekeeping gene. The TRNzol reagent extracted total RNA of HT-29 cells, and cDNA was synthesized using the PrimeScript™ reverse transcriptase (RT) reagent kit (Perfect Real Time) according to specification. The PCR reactions were performed using GoTaq^®^ qPCR Master Mix on a QuantStudio™ 3 Real-Time PCR System (Applied Biosystems, United States). The PCR primers are shown in Supplementary Table 1. Fold changes between the different groups were calculated using the 2^–ΔΔ^ cycle threshold method (Livak and Schmittgen, 2001).

### Statistical Analysis

All data were expressed as the mean ± standard deviation (SD). Data were analyzed using SPSS 16.0 software (SPSS Inc., Chicago, IL, United States). The statistical significance of data comparisons among the various groups was determined using one-way analysis of variance, followed by Duncan multiple-range test; *t*-test was used to compare the difference between the two groups. *p* < 0.05 was considered to be statistically significant.

## Results

### Effect of *Lactobacillus acidophilus* KLDS1.0901 on Cell Viability

**FIGURE 1:**
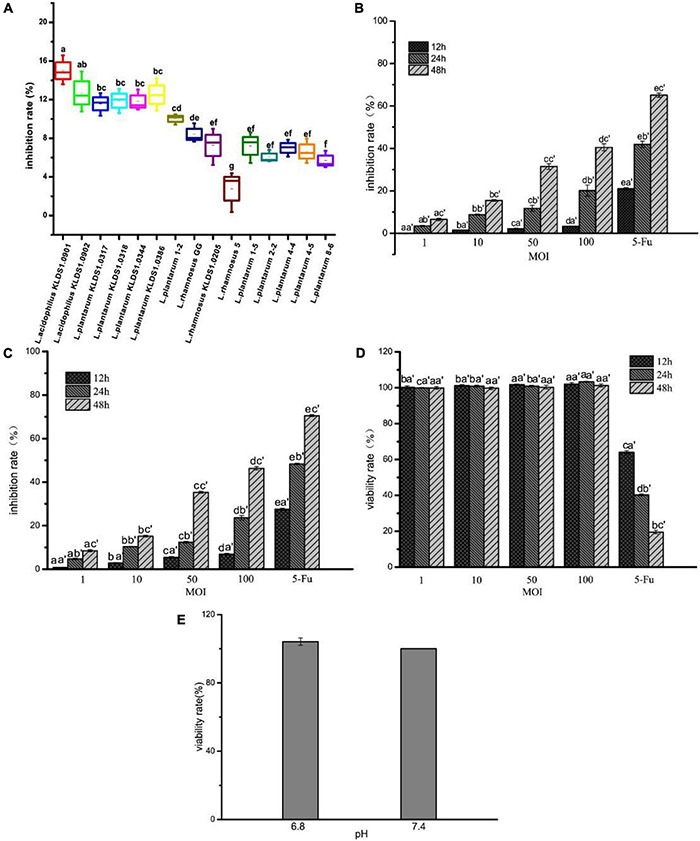
Effect of 15 strains on the inhibition rate of HT-29 cells **(A)**. Effect of *L. acidophilus* KLDS1.0901 on the inhibition rate of HT-29 cells **(B)**, Caco-2 cells **(C)**, and the viability rate of IEC-6 cells **(D)**. The viability rate of HT-29 cells in different pH of DMEM cell culture medium **(E)**. Treatment of HT-29 cells with 15 strains MOI = 10 for 48 h. Treatment of HT-29 cells, Caco-2 cells and IEC-6 cells with *L. acidophilus* KLDS1.0901 of different MOI (1, 10, 50, 100) and 5-FU (100 μg/mL) of treatment in 12, 24, and 48 h. Treatment of HT-29 cells with pH 6.8 and 7.4 of DMEM cell culture medium for 48 h. All data are presented as means ± SD (*n* = 3). Different lowercase letters (a–f) above the columns indicate significant data differences between different groups (*p* < 0.05), (a’, b’, and c’) indicate significant data differences between the same group.

The inhibition rate of 15 strains on HT-29 cells, *L. acidophilus* KLDS1.0901 and on HT-29 and Caco-2 cells, and the viability rate of *L. acidophilus* KLDS1.0901 on IEC-6 cells are shown in Figure 1. All 15 strains showed the inhibitory effect on HT-29 cells, and the inhibition rate was 2.78% to 15.00%. Among them, the inhibition rate of seven strains was higher than that of *L. rhamnosus* GG and was significantly higher than the remaining strains (*p* < 0.05). The inhibition rate of *L. rhamnosus* KLDS1.0205 and 5 *L. plantarum* numbered 1–5, 2–2, 4–4, 4–5, and 8–6 were all significantly lower than that of *L. rhamnosus* GG (*p* < 0.05). The inhibition rate of *L. acidophilus* KLDS1.0901 was the highest, and *L. rhamnosus* 5 was the lowest among the 15 strains (Figure 1A). HT-29 cells treated with *L. acidophilus* KLDS1.0901 showed a significant (*p* < 0.05) reduction of cell proliferation compared with nontreated cells. Importantly, *L. acidophilus* KLDS1.0901 inhibited the growth of HT-29 cells in a dose-dependent manner. *L. acidophilus* KLDS1.0901 exhibited a significantly lower inhibition rate on HT-29 cells at 12 h than at 24 and 48 h in the same MOI (*p* < 0.05), whereas 5-FU showed 21.00%. However, there was almost no inhibitory effect when MOI = 1 at different times, and the max inhibition rate was 40.51% when MOI = 100 at 48 h (Figure 1B). Meanwhile, the inhibition rate of Caco-2 cells was also dose-dependent. It was a significant (*p* < 0.05) reduction of cell proliferation at different times in the same MOI (*p* < 0.05). The inhibition rate reached the max when MOI = 100 at 48 h (Figure 1C). There was no significant difference (*p* > 0.05) in the viability rate of the same MOI at 12 h, 24, and 48 h on IEC-6 cells. On the contrary, 5-FU was a significant difference (*p* < 0.05) at 12, 24, and 48 h (Figure 1D). These results indicated that *L. acidophilus* KLDS1.0901 inhibited the growth of HT-29 and Caco-2 cells, and the viability rate of IEC-6 cells was not affected. Therefore, *L. acidophilus* KLDS1.0901 has no toxic effect on IEC-6 cells. The DMEM cell culture medium of pH 6.8 and pH 7.4 were used to culture the HT-29 cells (Figure 1E). The viability rate of HT-29 cells was not significantly different (*p* > 0.05) on pH 6.8 and 7.4. Therefore, the pH of DMEM cell culture medium had no effect on cell activity. At the same time, we focused on the pH of DMEM cell culture medium during *L. acidophilus* KLDS1.0901 treated with HT-29 cells. The initial pH of the DMEM cell culture medium in this experiment was 7.9; the final pH of DMEM cell culture medium slightly decreased to 7.1 after *L. acidophilus* KLDS1.0901 treated with HT-29 cells at MOI = 100 for 48 h. The culture medium pH 7.1 to simulate the adhesion of *L. acidophilus* KLDS1.0901 to cancer cells may be consistent with the real environment in the body. Then, we further studied the Hoechst staining, apoptosis, ROS level, and mitochondrial membrane potential of HT-29 when MOI = 10, 50, and 100 at 24 and 48 h.

### Hoechst Staining Assay

**FIGURE 2:**
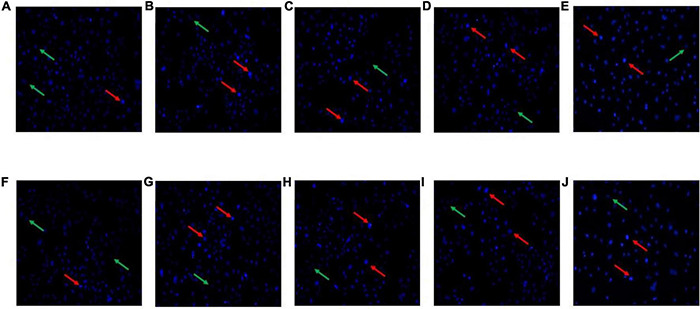
Morphological features of the HT-29 cells treated with *L. acidophilus* KLDS1.0901 of different MOI (0, 10, 50, and 100) and 5-FU (100 μmol/L) for 24 h **(A–E)** and 48 h **(F–J)**. A fluorescence microscope was used to photograph images (20×). The red and green arrows indicate the corresponding apoptosis and intact cells.

After Hoechst 33258 staining, under a fluorescence microscope, the nucleus of normal cells is ordinarily blue, whereas the nucleus of apoptotic cells will be densely stained, or clumpy densely stained, with a little whitish. The HT-29 cells treated with *L. acidophilus* KLDS1.0901 were stained with Hoechst 33258, and results showed that HT-29 cells were readily discernible, small, morphologically uniform, and bright fluorescent bodies. However, the extranuclear background appeared uniformly dark in nontreated cells. In general, the blue fluorescence of HT-29 cells treated with high MOI was more substantial than that treated with low MOI simultaneously, especially when MOI = 100 at 48 h. In comparison with 24 h, the nucleus was more densely stained after the *L. acidophilus* KLDS1.0901 treatment at 48 h (Figure 2). This phenomenon indicated that *L. acidophilus* KLDS1.0901 induced the apoptosis of HT-29 cells.

### Assaying of Apoptosis

**FIGURE 3:**
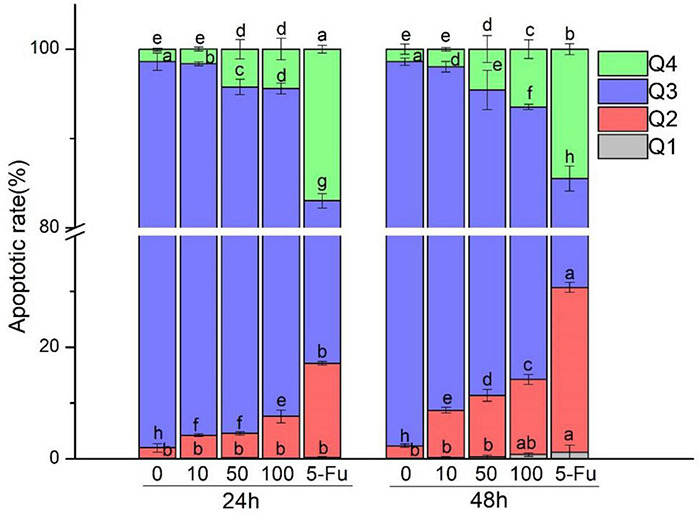
Effect of *L. acidophilus* KLDS1.0901 on apoptosis of HT-29 cells. *L. acidophilus* KLDS1.0901 were incubated in HT-29 cells of different MOI (0, 10, 50, and 100) and 5-FU (100 μmol/L) for 24 and 48 h. Q1–Q4 represents necrotic cells, late apoptotic cells, nonapoptotic cells, and early apoptotic cells.

The results of fluorescence microscopy alone cannot convincingly detect apoptosis. Therefore, we further detected the apoptosis rate. The results are shown in Figure 3, and Table 1 demonstrates that the portion of apoptotic cells (late and early late apoptotic cells, Q2 and Q4) was enhanced with the increase in MOI. HT-29 cells treated with *L. acidophilus* KLDS1.0901 and 5-FU showed a significant (*p* < 0.05) increase in apoptotic cells compared with nontreated cells. The positive control group (5-FU) increased the apoptosis rate (Q2 and Q4) from 33.8 to 44.03% at 24 and 48 h, respectively. *L. acidophilus* KLDS1.0901 revealed a significantly higher apoptotic rate in HT-29 cells at 48 h than that at 24 h in the same MOI (*p* < 0.05); the apoptosis rate of those treated with *L. acidophilus* KLDS1.0901 was lower than 5-FU. The maximum rate of apoptosis (Q2 and Q4) at 48 h reached 19.9%. Therefore, it had a more significant impact on apoptosis than nontreated cells with increased MOI. Then, we detected the changes in ROS level and mitochondrial membrane potential caused by apoptosis.

**TABLE 1:** Effect of *L. acidophilus* KLDS1.0901 on apoptosis of HT-29 cells.

Time (h)	Stage	MOI				5-FU (100 μg/mL)
		0	10	50	100	
24	Q1	0.10 ± 0.10^b^	0.10 ± 0.00^b^	0.20 ± 0.10^b^	0.17 ± 0.06^b^	0.30 ± 0.01^b^
	Q2	1.90 ± 0.79^h^	4.13 ± 0.23^f^	4.37 ± 0.32^f^	7.47 ± 1.16^e^	16.83 ± 0.35^b^
	Q3	96.6 ± 0.98^a^	94.13 ± 0.23^b^	91.2 ± 0.87^c^	87.97 ± 0.60^d^	65.9 ± 0.82^g^
	Q4	1.37 ± 0.15^e^	1.67 ± 0.21^e^	4.23 ± 1.09^d^	4.4 ± 1.20^d^	16.97 ± 0.42^a^
48	Q1	0.10 ± 0.10^b^	0.23 ± 0.15^b^	0.37 ± 0.38^b^	0.8 ± 0.30^ab^	1.20 ± 1.30^a^
	Q2	2.27 ± 0.32^h^	8.5 ± 0.50^e^	11.03 ± 1.07^d^	13.43 ± 0.90^c^	29.53 ± 0.91^a^
	Q3	96.23 ± 0.40^a^	89.3 ± 0.6^d^	84.03 ± 2.19^e^	79.3 ± 0.3^f^	54.76 ± 1.40^h^
	Q4	1.4 ± 0.56^e^	1.97 ± 0.21^e^	4.57 ± 1.50^d^	6.47 ± 1.05^c^	14.5 ± 0.62^b^

*Different superscript letters (a–h) indicate significant data differences between the same stage (p < 0.05). Q1–Q4 represent necrotic cells, late apoptotic cells, nonapoptotic cells, and early apoptotic cells.*

### Assaying of Intracellular Reactive Oxygen Species and Mitochondrial Membrane Potential

**FIGURE 4:**
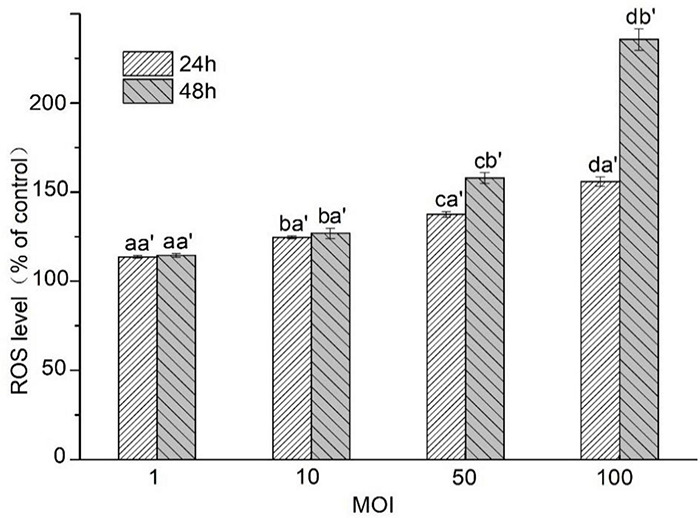
Effects of *L. acidophilus* KLDS1.0901 on intracellular ROS production of HT-29 cells. HT-29 cells were treated with *L. acidophilus* KLDS1.0901 of different MOI (1, 10, 50, and 100) for 24 and 48 h. All data are presented as means ± SD (*n* = 3). Different lowercase letters (a, b, c, and d) above the columns indicate significant data differences between different groups (*p* < 0.05), a’ and b’ indicate significant data differences between the same group.

The ROS level in HT-29 cells treated with *L. acidophilus* KLDS1.0901 of different MOI at 24 and 48 h was assayed. As shown in Figure 4, the ROS level in HT-29 cells was enhanced gradually along with MOI’s increase at 24 and 48 h. However, it showed a higher ROS level at 48 h than 24 h (*p* < 0.05) at the MOI = 50 and MOI = 100. It was noted that the maximum ROS level (235.59% ± 6.04%) in HT-29 cells was observed at 48 h (MOI = 100). These results indicated that *L. acidophilus* KLDS1.0901 induced ROS accumulation in HT-29 cells due to the apoptosis of the cells.

**FIGURE 5:**
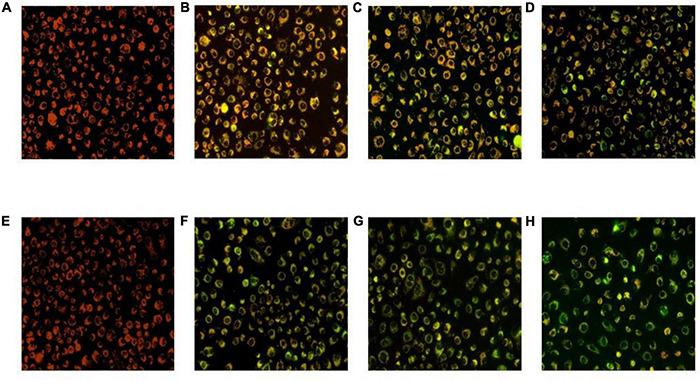
Mitochondrial membrane potential of the HT-29 cells treated with *L. acidophilus* KLDS1.0901. *L. acidophilus* KLDS1.0901 treated with HT-29 cells of different MOI (10, 50, and 100) for 24 h **(A–D)** and 48 h **(E–H)**. A fluorescence microscope was used to photograph images (20×).

The HT-29 cells treated with *L. acidophilus* KLDS1.0901 were observed using the JC-1 staining and fluorescence microscope. Green fluorescence indicates a decrease in mitochondrial membrane potential, and the cells are likely to be in the early stages of apoptosis. Red fluorescence shows that the mitochondrial membrane potential of cells was normal. It was observed that HT-29 cells treated with *L. acidophilus* KLDS1.0901 produced noticeable green fluorescence with MOI’s increase at 24 and 48 h (Figure 5). Meanwhile, it indicated that the rate of green to red fluorescence was higher at 48 h compared with 24 h (*p* < 0.05) at MOI = 10, 50, and 100, and the maximum rate of green to red fluorescence was 12.26 when MOI = 100 at 48 h (Table 2). These results suggested that *L. acidophilus* KLDS1.0901 reduced mitochondrial membrane potential and led to the apoptosis of HT-29 cells.

**TABLE 2:** Mitochondrial membrane potential ratio (green fluorescent/red fluorescent).

Time (h)	MOI			
	0	10	50	100
24 24	6.36 ± 0.21^a,a’^	7.38 ± 0.03^b,a’^	8.20 ± 0.13^c,a’^	9.06 ± 0.01^d,a’^
48	6.23 ± 0.03^a,a’^	8.71 ± 0.04^b,b’^	9.58 ± 0.10^c,b’^	12.26 ± 0.04^d,b’^

*All data are presented as means ± SD (n = 3). Different superscript letters (a–d) indicate significant data differences between different groups (p < 0.05). a′ and b′ indicate significant data differences between the same group (p < 0.05).*

### Gene Differential Expression Analysis

**FIGURE 6:**
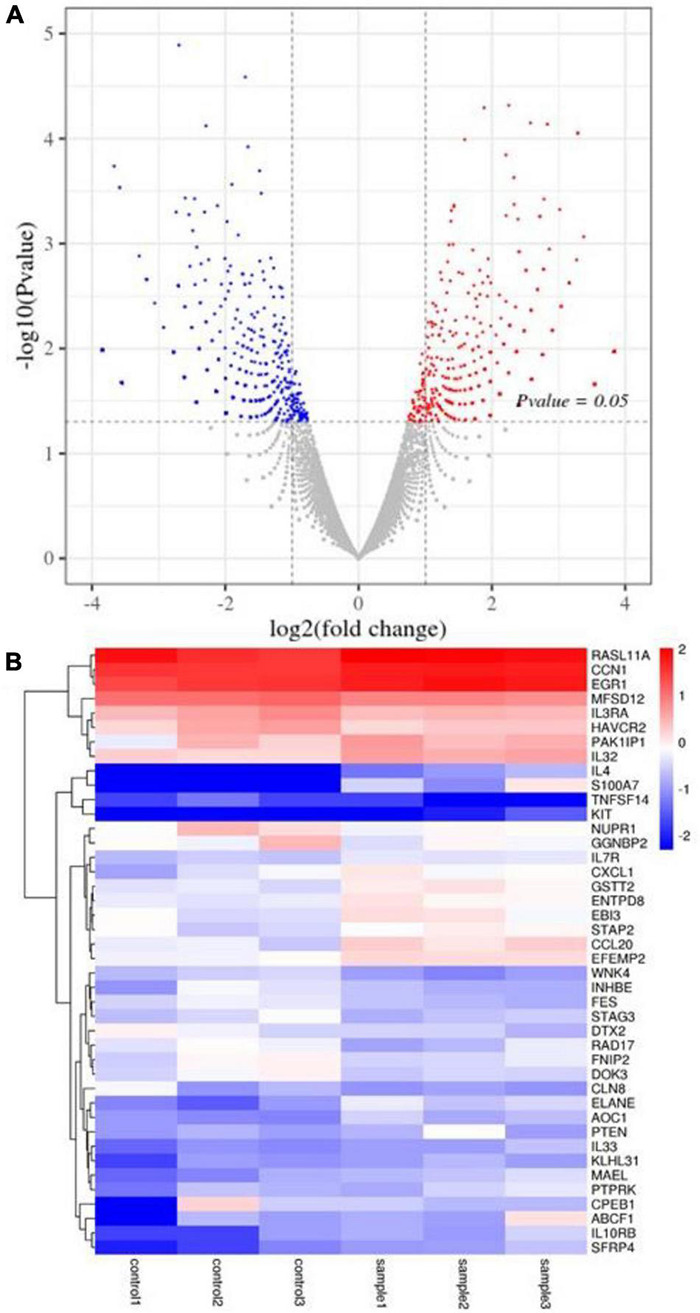
DEG analysis of HT-29 cells treated with *L. acidophilus* (*n* = 3). **(A)** The volcano plot of a differential gene. **(B)** Clustering heatmap of differentially expressed gene expression. Controls 1, 2, and 3 were nontreated with *L. acidophilus* in HT-29 cells. Samples 1, 2, and 3 were treated with *L. acidophilus* of MOI 100 for 48 h in HT-29 cells. The red dots in the differentially expressed gene volcano map represent up-regulated, differentially expressed genes; blue dots represent down-regulated, differentially expressed genes, and gray dots represent non-differentially expressed genes. The heatmap expresses cancer-related genes, where red represents genes with high log2 (FPKM/average FPKM) expression, and blue represents low expression.

Here, 1,133 DEGs were screened between the sample group and the untreated control group in HT-29 cells. Among them, there were 531 up-regulated genes and 602 down-regulated genes (Figure 6A). Heatmap expression of cancer-related gene expression is shown in Figure 6B. It was observed that the *CXCL1*, *GSTT2*, *ENTPD8*, *EBI3*, *STAP2*, *CCL20*, and *EFEMP2* genes were lower in the control group and higher in the sample group. The expression levels of *RASL11A*, *CCN1*, *EGR1*, *MFSD12*, *IL3RA*, *HAVCR2*, *PAK1IP1*, and *IL32* genes were higher in the sample group and control group. However, the sample group’s *RASL11A*, *CCN1*, and *EGR1* genes were higher than those of the control group. The expression levels of genes such as *IL4*, *S100A7*, *TNFSF14*, and *KIT* were low, especially in the control group. The expression levels of *WNK4*, *INHBE*, *FES*, *STAG3*, *DTX2*, *RAD17*, *FNIP2*, *DOK3*, and *CLN8* genes in the sample group were lower than those in the control group; that of the sample group including *ELANE*, *AOC1*, *PTEN*, *IL33*, *KLHL31*, *MAEL*, *PTPRK*, *CPEB1*, *ABCF1*, *IL10RB*, and *SFRP4* genes in the control group was less (Figure 6B).

### Gene Ontology Enrichment Analysis of Differentially Expressed Genes

**FIGURE 7:**
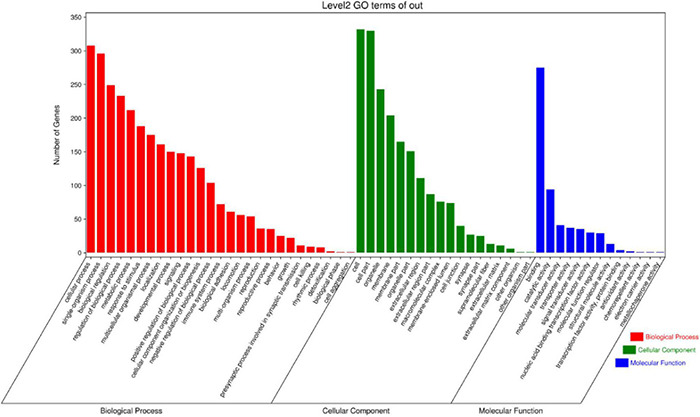
GO classification of differentially expressed genes.

Differentially expressed genes (DEGs) participated in 552 cell components, 4,952 biological processes, and 898 molecular functions (Figure 7). According to the molecular function, cell composition, and biological process, genes are classified by GO function enrichment analysis. From these two samples, the biological processes with significantly different enrichment were screened, including cellular process, single-organism process, biological regulation, regulation of biological process, and metabolic process. Significantly enriched cell component processes include cells, cell part, organelle, membrane, and membrane parts. The molecular functions with significantly different enrichment include binding, catalytic activity, molecular transporter activity, transporter activity, and signal transducer activity.

### Kyoto Encyclopedia of Genes and Genomes Enrichment Analysis of Differentially Expressed Genes

**FIGURE 8:**
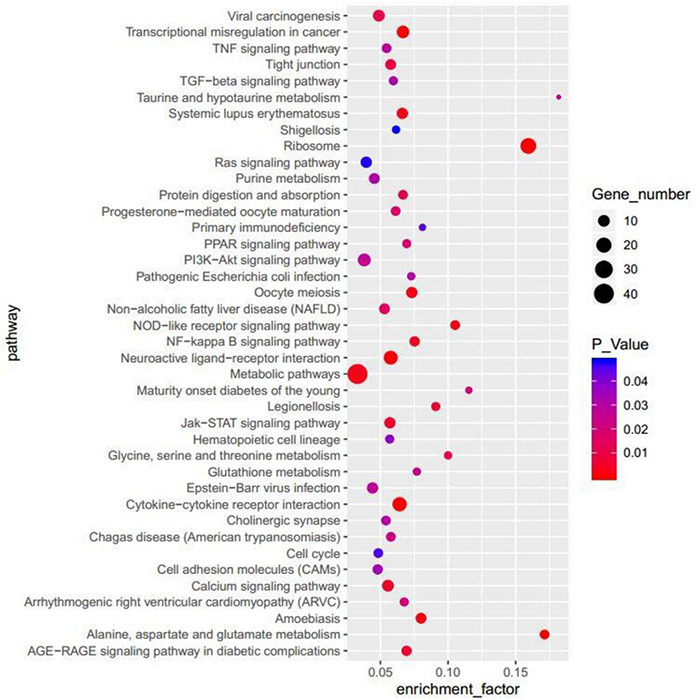
The top 40 terms enriched in the KEGG of the DEGs.

For further analysis, we used the KEGG enrichment method to identify specific pathway terms, whereas 1,133 DEGs were enriched in 248 KEGG pathways. There were top 40 pathways with *p* < 0.05 (Figure 8). Among those associated with cancers: overviews were transcriptional misregulation in cancer and viral carcinogenesis; related to cell growth and death were oocyte meiosis and cell cycle; connected with immune diseases and immune system were systemic lupus erythematosus, primary immunodeficiency, NOD-like receptor signaling pathway, and hematopoietic cell lineage; correlated with signal transduction were nuclear factor κB (NF-κB) signaling pathway, calcium signaling pathway, Jak-STAT signaling pathway, PI3K-Akt signaling pathway, tumor necrosis factor (TNF) signaling pathway, transforming growth factor β signaling pathway, and Ras signaling pathway; and connected with signaling molecules and interaction were neuroactive ligand–receptor interaction and cell adhesion molecules. This study focused on the NF-κB signaling pathway, calcium signaling pathway, and PI3K-Akt signaling pathway.

### Predictive Analysis of Protein Interactions

**FIGURE 9:**
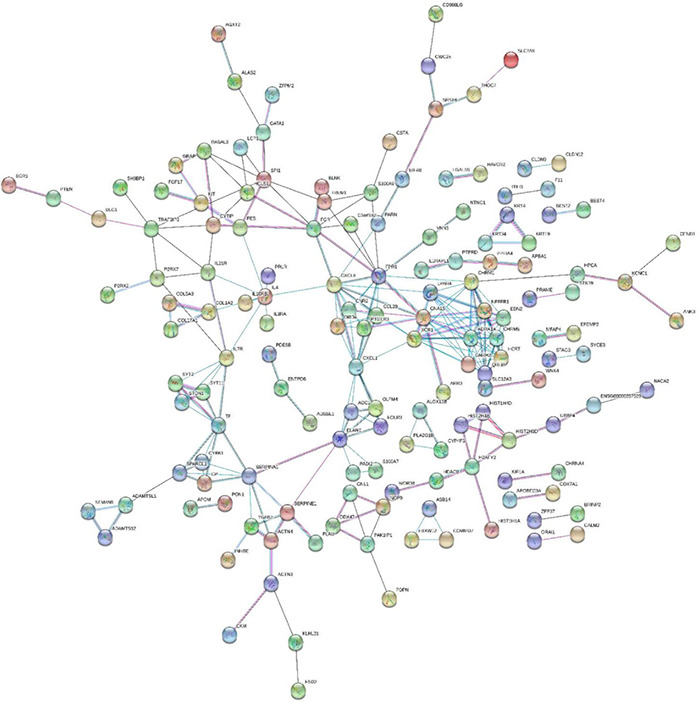
Diagram of protein–protein interactions predicted by string.

The search tool (STRING) combined with 150 DEGs was used to search for interacting genes (Szklarczyk et al., 2016), and PPIs were generated (Figure 9). In this network, *ADRA1A*, *CHRM5*, *CXCL1*, *ELANE*, *FGR*, *FPR1*, *LPAR4*, *QRFP*, and *SERPINA1* were counted to have high connectivity degree of each node, in which *FPR1* was the highest. To this end, we identified *FPR1*, *CXCL1*, and *ELANE* as the hub genes. Also, *FPRI* needed to correlate with *CHRM1* in our study. Protein–protein interaction was used to predict the mechanism of *Lactobacillus*-induced colon cancer for further research.

### Verification of Transcriptome Sequencing Results by Real-Time Quantitative Polymerase Chain Reaction

**FIGURE 10:**
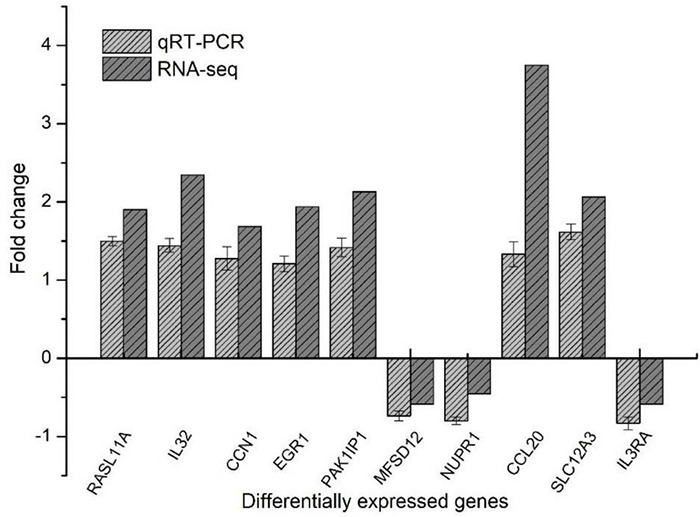
RT-qPCR to verify differentially expressed genes.

The RT-qPCR experiment was performed to verify seven up-regulated and three down-regulated DEGs of HT-29 cells treated with *L. acidophilus* KLDS1.0901. As shown in Figure 10, although the changed folds of DEGs obtained by RT-qPCR were different from RNA-seq, the gene expression trend was the same. Our findings revealed the reliability of the transcriptomics results.

## Discussion

*Lactobacillus* is a group of microorganisms beneficial to the host’s health, and they play a probiotic effect mainly through colonization in the intestine. It was reported that specific *Lactobacillus* could confer health benefits to the colon. Cytotoxicity studies focus on the antiproliferative and apoptosis effects of *Lactobacillus* in various cell lines *in vitro* (Orlando et al., 2012). At the same time, studies have also shown that probiotics in animal models have inhibitory activity against the colon *in vivo* (Reddy and Rivenson, 1993). The control strain *L. rhamnosus* GG used in this experiment has a potential protective effect on colon cancer and could induce apoptosis and reduce inflammation (Gamallat et al., 2016). When Wang et al. (2014) initially screened LABs, the inhibition rate of HT-29 cells was 5% to 32% under the conditions of MOI = 10 at 48 h, and most of them were more than 10%, and consistent with our results. Therefore, *L. acidophilus* KLDS1.0901 had the highest inhibition rate on HT-29 cells and was selected for subsequent experiments. A study investigated the inhibitory effect of probiotic *Lactobacillus* and supernatants on the growth of HT-29 cells, and seven studies of *Lactobacillus* have different apoptosis effects on HT-29 cells (Chen et al., 2017). The survival rate of colon cancer cells was significantly reduced to 78% *in vitro* when *L. casei* ATCC 393 of 10^9^ CFU/mL was incubated with HT-29 cells for 24 h (Tiptiri-Kourpeti et al., 2016). Meanwhile, our results showed that *L. acidophilus* KLDS1.0901 also diminished proliferation, supporting previous observations. Guo et al. (2020) found that the *L. acidophilus* CICC 6074 significantly inhibited cell proliferation in a time- and dose-dependent manner. When the bacteria with a concentration of MOI = 100 treated HT-29 cells for 48 h, the inhibition rate reached 57.26% ± 2.21%. It was consistent with our experimental results. A study found that *L. plantarum* 06CC2 had an inhibitory effect of the extract on the growth of Caco-2 cells *in vitro* (Hiraishi et al., 2019). It indicated that the extract of *Lactobacillus* had a cancer-inhibition effect. Hasan (2021) studied the cell proliferation and inhibition rate by using different concentrations of *L. acidophilus* growth extracts on Caco-2 cells. It shows that the extracts of strains LB3 and LB5 have high cytotoxicity on Caco-2 cells with IC50 40.8 and 51 μg/mL, respectively. Cancer cells have a lower extracellular pH of 6.7–7.1 and a higher intracellular pH of 7.4, whereas normal cells have a higher extracellular pH of 7.4 and a lower intracellular pH of 7.2 (Lee and Shanti, 2021). Li et al. detected the colon cancer cell of SW116 in the extracellular pH 6.8 (pHe6.8) environment; the growth of tumor cells was more vigorous, and their vitality was significantly increased compared with pHe7.4 (Li et al., 2014). Similarly, DMEM cell culture medium with different pH had no toxic effect on HT-29 cells. In this experiment, the final pH was slightly decreased to 7.1, and *L. acidophilus* can still inhibit proliferation. These prove that *Lactobacillus* may have inhibited proliferation effects in cancer cells, and the inhibition effect might be due to the tumor cells’ biological changes.

Hoechst can stain a concentrated nucleus to distinguish apoptosis cells from normal and necrotic cells (Wu et al., 2019). As previously reported, all control cells were found to be intact and in a normal state after Hoechst staining, whereas cells treated with 5-FU and secretion metabolites of *Kluyveromyces marxianus* AS41 showed a contraction of cells’ volume, an early stage of apoptosis, or late stage of apoptosis nuclear (Saber et al., 2017). HT-29 cells treated with *L. acidophilus* showed morphological changes of apoptosis characteristics, including chromatin condensation, whereas untreated cells showed dark images of cells.

In 1995, annexin V was used to detect apoptosis. Different cell types exert phosphatidylserine to the cell surface (Vermes et al., 1995). Wang et al. (2014) found that the cell wall extracts of *Lactobacillus* X12, M5, and K14 increased the early stage of apoptosis rate by 0.51% and the late apoptosis stage 0.11%–4.27% compared with LGG on HT-29 cells through attachment V–FITC staining assay. Consistent with the literature, this research found that *L. acidophilus* KLDS1.0901 also increased early apoptosis and late apoptosis rates.

Excessive ROS production has been implicated in many diseases because it can cause oxidative stress. Probiotics can prevent the inflammatory response of cultured epithelium by inducing ROS production. Moreover, the massively generated ROS aggravated the damage of mitochondria and apoptosis (Haynes et al., 2004). Xiao et al. (2020) found that one fraction of LHEPS-1 isolated from exopolysaccharide in *Lactobacillus helveticus* MB2-1 could enhance intracellular production of ROS. Our study supported evidence from previous observations that the increase in ROS production of *L. acidophilus* KLDS1.0901 treated with HT-29 cells was positively correlated with the increase in apoptosis at 24 and 48 h, which was reasonable.

In general, the decrease in mitochondrial membrane potential is an important sign of the early stage of apoptosis and also increases the release of cytochrome c and activates caspase-3, caspase-8, and caspase-9 (Liu et al., 2011). Mitochondria in the death of Caco-2 cells induced by lipid-rich extract from Mexican avocado showed that after 6 h, treatment with LEAS could lead to a loss of 58.2% of mitochondrial membrane potential (Sánchez-Pérez et al., 2009). In this study, there were some similarities that *L. acidophilus* KLDS1.0901 could increase green fluorescence (the decrease in mitochondrial membrane potential). Therefore, the reduction in cell membrane potential can be easily detected by the transition from red fluorescence to green fluorescence of JC-1. In addition, the transition from red fluorescence to green fluorescence of JC-1 can also be used as an indicator of early stage of apoptosis.

In this study, transcriptomics technology was used to analyze the DEGs of *L. acidophilus* KLDS1.0901 in decreasing apoptosis of HT-29 cells. *MFSD12* plays an important role in moving compounds of biofilms and is associated with various cancers. The increased *MFSD12* expression promoted melanoma cells’ proliferation, indicating that *MFSD12* mRNA may be a new diagnostic target with high sensitivity and specificity (Wei et al., 2019). *5-NUPR1* (nucleoprotein 1) plays a crucial role in developing many malignant tumors. The down-regulation of endogenous *NUPR1* inhibitors can reduce lung cancer cells’ proliferation, leaving the cells in the G0/G1 cycle and leading to apoptosis (Guo et al., 2012). In this experiment, the expression of *MFSD12* and *NUPR1* mRNA in the *L. acidophilus* KLDS1.0901 treatment group was lower than that of nontreated cells, indicating that low *MFSD12* and *NUPR1* are related to inhibiting cancer cell proliferation and promoting apoptosis. Miene et al. (2011) studied that polyphenols may play an important role in the prevention of colon cancer. The metabolites of intestinal microbiota do not affect the number of cells, but significantly increase the expression of *GSTT2* and decrease the expression of *COX-2*. The increased *GSTT2* and the decreased *COX-2* may contribute to the chemopreventive potential of polyphenols after intestinal degradation. The *ENTPD* family may be associated with inflammation and ROS, partly associated with cancer. The metabolism of CTP into dCTP, apoptosis, and inhibiting proliferation may be promoted because of the overexpression of *ENTPD8* (An et al., 2018). The above studies are consistent with the increased expression of *GSTT2* and *ENTPD8* after treating with *L. acidophilus* KLDS1.0901, indicating that *GSTT2* and *ENTPD8* were closely related to colon cancer.

Calcium is a ubiquitous intracellular ion that acts as a signaling mediator in numerous cellular processes, including cell proliferation, differentiation, and survival/death. In the past study, cadmium toxicity was partially due to its disruption of intracellular Ca^2+^ homeostasis by compromising ATPase activities and ER-regulated Ca^2+^, and this elevation in Ca^2+^ triggers the activation of the Ca^2+^–mitochondria apoptosis signaling pathway (Yuan et al., 2013). A study showed that uridine triphosphate could indirectly increase intracellular Ca^2+^ levels, inhibit hepatic stellate cell proliferation, and promote apoptosis through the CaMK II/Ca^2+^ signaling pathway (Liu et al., 2021), which was accorded with our study that *L. acidophilus* KLDS1.0901 also activated calcium signaling pathway. The PI3K-Akt signaling pathway plays a critical role in mediating survival signals in many neuronal cell types. It may be antagonized by the tumor suppressor phosphatase and tensin homolog (*PTEN*). AKT’s central role in the PI3Ks pathway makes it one of the most activated downstream effectors in the oncogenic landscape (Martini et al., 2014). Huang et al. found that gigantol could inhibit the proliferation and significantly potentiate the anticancer activities of cisplatin (DDP) in breast cancer cells via the PI3K/Akt/mTOR signaling pathway (Huang et al., 2021). In our study, *L. acidophilus* KLDS1.0901 affected apoptosis through key genes of *PTEN* and *AKT* in the PI3K-Akt signaling pathway. NF-κB signaling pathway in immunoregulatory functions is well accepted. Activating the NF-κB signaling pathway leads to the induction of target genes that can interfere with apoptosis and the cell cycle. Jani et al. (2010) studied that quinacrine abrogates NF-κB activation and stimulates apoptosis by increasing the cytotoxicity of TNF-related apoptosis-inducing ligand in RKO and HT-29 cells. *Lactobacillus acidophilus* KLDS1.0901 could induce the NF-κB signaling pathway through *NIK* and *TRAF6*. Moreover, Maeda et al. (2000) reported that *Helicobacter pylori* induces NF-κB activation through an intracellular signaling pathway that involves *IKKα*, *IKKβ*, *NIK*, *TRAF2*, and *TRAF6*.

Formyl peptide receptor 1 (*FPR1*) is a G protein–coupled receptor, mainly expressed by bone marrow–derived cells, and it mediates the innate immune response to bacteria-forming peptides. It has been studied that inhibiting *FPR1* activity in NB cells decreases tumor growth in xenograft models; however, overexpression increases tumor burden (Snapkov et al., 2016). Also, muscarinic acetylcholine receptors mediate cellular responses and multiple responses, involving the regulation of potassium channels. Knockout of M1 muscarinic receptor (*CHRM1*) in azomethane-treated mice can reduce the number and size of colon cancer (Cheng et al., 2010). It indicated that the down-regulation of *FPR1* and *CHRM1* in this study might relate to the decreasing growth of HT-29 cells.

## Conclusion

*L. acidophilus* KLDS1.0901 could inhibit the proliferation of HT-29 and Caco-2 cells, which depended on the dose of MOI and time. The maximum inhibition rate was reached at 48 h and MOI = 100. It also increased ROS level and apoptosis rate and decreased the mitochondrial membrane potential of HT-29 cells. Transcriptomic sequencing analysis showed that 1,133 DEGs were screened. Meanwhile, the DEGs were primarily enriched in NF-κB, PI3K-Akt, calcium, and other signaling pathways via KEGG analyses related to the apoptosis mechanism. Moreover, the study predicted all functional interactions between proteins expressed by crucial genes. Therefore, our findings provided a relatively comprehensive understanding of the proliferation and apoptosis of *Lactobacillus*.

## Data Availability Statement

The original contributions presented in the study are publicly available. This data can be found here: [https://www.ncbi.nlm.nih.gov/sra/PRJNA804959] SRR17965733, SRR17965734, SRR17965735, SRR17965736, SRR17965737, and SRR17965738.

## Author Contributions

BL and GH planned and supervised the experiments. YY and SW carried out the experiments and wrote the manuscript. YY, JS, QX, NL, and JG analyzed the data. YY and FL prepared the figures. SE revised the manuscript. All authors have read and agreed to the final manuscript draft.

## Conflict of Interest

QX was employed by Heilongjiang Feihe Dairy Co., Ltd. The remaining authors declare that the research was conducted in the absence of any commercial or financial relationships that could be construed as a potential conflict of interest.

## Publisher’s Note

All claims expressed in this article are solely those of the authors and do not necessarily represent those of their affiliated organizations, or those of the publisher, the editors and the reviewers. Any product that may be evaluated in this article, or claim that may be made by its manufacturer, is not guaranteed or endorsed by the publisher.

## Footnotes

1
http://string-db.org/
