# Supplementary material for: Effects of Lactobacillus acidophilus KLDS1.0901 on Proliferation and Apoptosis of Colon Cancer Cells
Source: Front Microbiol. 2022 Feb 11;12:788040. doi: 10.3389/fmicb.2021.788040 (PMC8895954; doi:10.3389/fmicb.2021.788040)
Supplement: Supplementary file 2 [file Data_Sheet_1.docx]

**Supplementary materials**

**Table S1** Specific human gene primers used for real-time quantitative RT-PCR

| **Gene** | **Forward primer** | **Reward primer** |
| --- | --- | --- |
| **RASL11A** | AGGTCCACCCTGACTCTAAAGCC | GCTGAATACCGTCCTGTGTCTGC |
| **IL32** | TGCTTCCCGAAGGTCCTCTCTG | ACTGTCTCCAGGTAGCCCTCTTTG |
| **CCN1** | GCATTCCTCTGTGTCCCCAAGAAC | ACCCACTCCTCGCAGCACTG |
| **MFSD12** | TCTGCGTCCTGCTGTCCTTCC | CTGAGGTGGGAGATCTGTGTGGAG |
| **PAK1IP1** | GCTGGTCGCTGGTTGCTACG | AAGTCAGCCACAAGAGTCCATTGC |
| **EGR1** | GCTGGAGGAGATGATGCTGCTG | TGCTGCTGCTGCTGCTGTTG |
| **NUPR1** | AAGCTGCTGCCAACACCAACC | TGCCCCTCGCTTCTTCCTCTC |
| **CCL20** | GCTCCTGGCTGCTTTGATGT | GCCGTGTGAAGCCCACAATA |
| **SLC12A3** | GGCACCAGCAGCGAGAAGAAC | AGCCGCAGGTAGAGGATCACG |
| **IL3RA** | GGCGTCAACAGTACGAGTGTCTTC | GGAACTTTGAGAACCGCTGGAGAG |
